# The Worker Honeybee Fat Body Proteome Is Extensively Remodeled Preceding a Major Life-History Transition

**DOI:** 10.1371/journal.pone.0024794

**Published:** 2011-09-28

**Authors:** Queenie W. T. Chan, Navdeep S. Mutti, Leonard J. Foster, Sarah D. Kocher, Gro V. Amdam, Florian Wolschin

**Affiliations:** 1 Centre for High-Throughput Biology and Department of Biochemistry and Molecular Biology, University of British Columbia, Vancouver, Canada; 2 Arizona State University, School of Life Sciences, Tempe, Arizona, United States of America; 3 Pennsylvania State University, Department of Entomology, University Park, Pennsylvania, United States of America; 4 Department of Biotechnology, Chemistry, and Food Science, Norwegian University of Life Sciences, Aas, Norway; Boston University, United States of America

## Abstract

Honeybee workers are essentially sterile female helpers that make up the majority of individuals in a colony. Workers display a marked change in physiology when they transition from in-nest tasks to foraging. Recent technological advances have made it possible to unravel the metabolic modifications associated with this transition. Previous studies have revealed extensive remodeling of brain, thorax, and hypopharyngeal gland biochemistry. However, data on changes in the abdomen is scarce. To narrow this gap we investigated the proteomic composition of abdominal tissue in the days typically preceding the onset of foraging in honeybee workers.

In order to get a broader representation of possible protein dynamics, we used workers of two genotypes with differences in the age at which they initiate foraging. This approach was combined with RNA interference-mediated downregulation of an insulin/insulin-like signaling component that is central to foraging behavior, the *insulin receptor substrate* (*irs*), and with measurements of glucose and lipid levels.

Our data provide new insight into the molecular underpinnings of phenotypic plasticity in the honeybee, invoke parallels with vertebrate metabolism, and support an integrated and *irs*-dependent association of carbohydrate and lipid metabolism with the transition from in-nest tasks to foraging.

## Introduction

Many organisms undergo distinct shifts in life-history that are coupled to changes in their physiology. Examples include changes of sex in fish [1], parasite adaptations to different hosts [2], and behavioral changes following mating in social insects [3], [4]. Another prominent example is the transition from in-nest tasks to foraging in worker honeybees [5]. The life of honeybee workers is characterized by a profound, age-associated change in their behavioral phenotype. Young worker bees typically stay inside the nest where they feed, brood, tend to the queen, and maintain the hygiene of the colony. Thereafter, worker bees become foragers and leave the colony for daily trips to collect food [4]. This behavioral change is linked to structural and biochemical alterations across the body [5]. Thorax muscle physiology is modified in order to meet the demands of the strenuous foraging flights [6]. The protein composition of the hypopharyngeal glands, a tissue that plays a major role in honeybee nutrition, undergoes major morphological and transcriptional restructuring that is linked to the different roles performed by nest bees and foragers [7]–[9]. In addition, brain metabolism is altered in order to account for the dramatic change in task and environment [7], [8], [9].

Less is known about molecular changes in the abdomen, including the fat body. The invertebrate fat body functions in energy storage, utilization, and detoxification [10], which makes it comparable to vertebrate adipose tissue and liver [11], [12]. Studies on the fat body can have implications beyond the realm of insect science as they provide important insights into possible molecular causes for obesity and longevity that can be general across taxa [13], [14], [15].

The honeybee fat body is more developed in nest bees compared to forager bees [16], and a decrease in abdominal lipids has been shown to precede the onset of foraging [17]. These observations indicate that the biochemistry of the abdomen is profoundly remodeled during the transition from in-nest tasks to foraging. However, how such changes are brought about on a molecular level remains largely unknown. An evolutionarily conserved regulator of carbohydrate and lipid metabolism is insulin signaling. Knocking out a major player of the insulin signaling network, the insulin receptor substrate gene *irs*, in *Drosophila* and mice can cause elevated lipid levels and a change in glucose homeostasis [18], [19], [20], [21]. Recently, changes in the expression of insulin signaling pathway genes were linked to the onset of foraging [22], and downregulation of *irs* expression in the fat body was shown to influence food collection by increasing the forager's preference for pollen (protein source) [23]. The extent of the latter was found to be higher in bees of a genotype that starts foraging earlier in life [24]. Overall, changes in lipid metabolism and insulin signaling components appear to be associated with the distinction between behavioral phenotypes and are connected to the life-history transition in honeybees. However, the majority of proteins involved remains to be uncovered.

Therefore, we employed a proteomics approach in order to shed light on the proteomic plasticity of honeybee abdominal tissue in young workers. In parallel, we monitored abdominal lipid and hemolymph glucose levels. We used bees of two different genotypes that differ in their onset of foraging and RNA interference (RNAi) to perturb *irs* gene expression. Downregulation of *irs* expression was performed in both genotypes in order to test the robustness of the molecular response to the knockdown. Based on prior studies (see above), we hypothesized that: 1. The abundance of proteins involved in lipid synthesis should change in an age-dependent manner independent of genotype; 2. Lipid levels should be higher in the genotype that initiates foraging later in life, if they are linked to the onset of foraging. These levels should also be positively influenced by the downregulation of *irs* in both genotypes if the connection between *irs* expression and lipid metabolism is evolutionarily conserved.

Workers for this study were collected at 7, 9, and 11 days of age, which represents different stages in their developmental ontogeny as nest bees [4], [25], all typically preceding the onset of foraging [25], [26]. During this period, the hemolymph levels of VG (vitellogenin), a protein that influences behavior, change dramatically [27], as do other features like the proteomic composition of the hypopharyngeal glands [7] and the levels of juvenile hormone (JH), a hormone that influences behavioral maturation in honeybees [28], [29].

We reveal genotype, *irs* knockdown treatment, and age-dependent effects on the proteomic pattern of the abdomen, and we provide data that support a maturation-dependent adjustment of lipid metabolism in worker bees. We also show that lipid levels vary with genotype and knockdown treatment in a predictable way. In addition, our findings include data on the JH-degrading enzyme juvenile hormone esterase (JHE), VG, and other proteins with potential implications for metabolic biology and behavioral plasticity.

## Results and Discussion

### 1. Artificial selection for food collection preference and *irs* mRNA levels influence the proteomic pattern of the abdomen

This experiment aimed to reveal possible implications of genotype and downregulation of *irs* expression on the proteomic pattern in the honeybee worker abdominal tissue at 7 days of adult life. Both factors - genotype and *irs* knockdown treatment - influence honeybee behavior [23], [24], [30]. The study of their impact on proteomic patterns can thus aid in our understanding of the metabolic underpinnings of behavioral plasticity. The RNAi-mediated knockdown of *irs* expression in our study [Figure S1] was comparable to a previously published experiment, which showed behavioral effects of *irs* knockdown [23]. Honeybees representing all possible combinations of two standard genotypes (high vs. low pollen hoarding) and dsRNA (double stranded RNA) treatments (control *gfp* (green fluorescent protein) vs. *irs* dsRNA) were collected: CH: *gfp* control high pollen hoarding genotypes; KH: *irs* knockdown high pollen hoarding genotypes; CL: *gfp* control low pollen hoarding genotypes; KL: *irs* knockdown low pollen hoarding genotypes), and resulting samples were compared using a quantitative label-free approach carried out essentially as described before [31].

**Figure 1:**
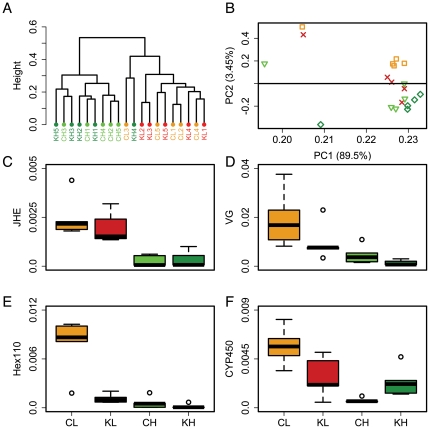
Genotype and manipulation of *irs* expression influence the proteomic pattern on a global and individual protein level. Color-coding: Orange: low pollen hoarding genotype control (CL), Red: low pollen hoarding genotype knockdown (KL); Light green: high pollen hoarding genotype control (CH); Dark green: high pollen hoarding genotype knockdown (KH). A: Hierarchical clustering analysis of log_2_-transformed abundance values of all quantifiable proteins from experiment 1; B: Principal components analysis based on the Pearson correlation matrix of all quantifiable proteins. Squares: low pollen hoarding genotype control, dagger: low pollen hoarding genotype knockdown, triangles: high pollen hoarding genotype control, diamonds: high pollen hoarding genotype knockdown; C, D, E, and F: Boxplots (medians and 25–75 percentiles) of corrected spectral count as a measure of abundance for juvenile hormone esterase (JHE), vitellogenin (VG), hexamerin 110 (Hex110), and cytochrome P450 monooxygenase (CYP450), respectively. Non-parametric Kruskal Wallis and Mann Whitney U tests (p<0.1, bootstrap verified cutoff) showed genotype effects on all four proteins and knockdown effects on VG, hexamerin 110, and cytochrome P450.

A hierarchical clustering analysis based on the log_2_-transformed data for all quantifiable proteins (147) from this experiment showed a clear separation of genotypes but overall failed to reveal effects of the knockdown [Figure 1A]. The exception was one high pollen genotype knockdown sample, which clustered with the low pollen genotype. Further, a principal components analysis corroborated the separation of the controls and revealed a more scattered pattern for the bees with lowered *irs* expression [Figure 1B]. This pattern is probably due to an increased variation in the knockdown groups compared to the control groups [Figure S2]. In this context, it should be noted that in previous studies, the foraging preference of high and low pollen hoarding genotype bees was mapped to quantitative trait loci (QTL, *pln*1–*pln*4), and *irs* was identified as a potential contributor to the phenotypic divide between the two genotypes [27]–[29]. Thus, however slight, an influence of *irs* expression on the proteomic differentiation between the two genotypes may not be surprising.

Overall, 49 protein species were found to differ in abundance between groups (Kruskal Wallis test, n = 5, each sample is a pool of proteins from 3 individual abdominal carcasses, p<0.1 bootstrap verified cutoff. Note that redundancy can occur when two or more proteins share the same peptides). Of these, 22 (17 non-redundant proteins) were affected by genotype when comparing the results for the control groups, 13 by treatment (combined for CL vs KL and CH vs KH), and 15 by treatment and genotype [Table 1, Table S1, and Figure S3] (Mann Whitney U-tests, n = 5, each sample is a pool of proteins from 3 individual abdominal carcasses, p<0.1 bootstrap corrected cutoff). Differences identified as genotype effects covered 10 proteins with higher abundance in CH vs CL (5 myosin-related proteins, pericardin, troponin C and T, a histone 2A-like protein, and an amine oxidase) and 7 proteins with higher abundance in CL vs CH (RFABP (retinoid and fatty acid binding protein), alpha tubulin, JHE, ribosomal protein 7, an oxidoreductase, a lon protease-like protein, and pugilist protein). The higher abundance of 8 muscle-related proteins (5 myosin-related, pericardin, troponin C and T) in CH indicates a stronger investment in the building blocks of abdominal muscle tissue in this genotype at 7 days of age, which could relate to the earlier onset of foraging compared to CL bees. Of the proteins that showed higher abundance in CL, we may speculate about the implications of RAFBP and JHE. A prime area of interest in this study is lipid metabolism, as higher lipid levels appear to be associated with the nest-bee stage. Since low pollen genotype bees start foraging later in life, we assumed that the abundance levels of proteins involved in lipid metabolism would differ between the two genotypes. Indeed, we found higher levels of a retinoid and fatty acid binding protein in the low pollen genotype bees. RAFBPs function in the binding and transportation of lipids as well as in lipid signaling [32]. Higher levels of RAFBP could indicate a more active lipid production/allocation in the CL group, which is in line with the proposed important role of lipid metabolism in the division of labor. In worker honeybees, JH is a key determinant of the switch from in-nest tasks to foraging, with higher levels being associated with the latter [33]. The higher abundance of the JH-degrading enzyme JHE in bees of the low pollen hoarding genotype [Figure 1 C] potentially explains why young bees of this genotype have lower JH levels than age-matched bees of the high pollen hoarding genotype [34]. JH is proposed to act as a repressor of VG synthesis [35], [36]. If the rate of JH synthesis in the two genotypes is the same (or lower in bees of the low pollen genotype) and degradation through JHE is higher in the low pollen genotype, we would expect lower JH levels and consequently higher VG levels in CL vs CH. VG levels are indeed higher in the low pollen hoarding genotype, as revealed in the proteins that were affected by genotype and treatment [Figure 1D]. The present observations of higher abdominal VG and JHE levels in bees of the low pollen hoarding genotype are in line with the postulated importance of VG and JH in regulating the onset of foraging [35]. A positive influence of VG on JHE expression could explain the previously observed negative impact of high VG levels on JH titers.

**Table 1:** Genotype and treatment-dependent differences in protein abundance as revealed by a label-free proteomics approach.

Accession #	Similar to	Effect	p-value CL/KL	RS1 CL	RS2 KL	p-value CH/KH	RS3 CH	RS4 KH	p-value CL/CH	RS5 CL	RS6 CH
XP_392490	Retinoid- and fatty-acid binding protein	G	0.151	35	20	0.222	21	34	0.008	40	15
XP_394991	Tubulin alpha-6 chain	G	1.000	27	28	0.222	21	34	0.008	40	15
NP_001011563	Juvenile hormone esterase	G	0.421	32	23	0.310	22	33	0.008	40	15
XP_001120641	Pericardin	G	0.222	21	34	0.841	29	26	0.008	15	40
XP_624700	Histone 2 A	G	1.000	28	27	1.000	28	27	0.008	15	40
XP_001120934	Histone 2 A	G	1.000	28	27	1.000	28	27	0.008	15	40
XP_001119899	Histone 2 A	G	1.000	28	27	1.000	28	27	0.008	15	40
XP_001120346	Histone 2 A	G	1.000	28	27	1.000	28	27	0.008	15	40
XP_001120186	Histone 2 A	G	1.000	28	27	1.000	28	27	0.008	15	40
XP_623470	Tropomyosin 1, isoform B isoform 2	G	0.690	30	25	0.690	30	25	0.016	16	39
XP_624943	Ribosomal protein S7, isoform A	G	0.310	33	22	0.841	26	29	0.016	39	16
XP_393281	Paramyosin, isoform A	G	0.421	23	32	1.000	28	27	0.016	16	39
XP_392125	Tropomyosin 1, isoform D isoform 1	G	0.690	30	25	1.000	28	27	0.016	16	39
NP_001011651	Troponin C type IIIa	G	0.690	25	30	0.690	30	25	0.032	17	38
XP_623046	Tropomyosin 1, isoform D	G	0.548	31	24	0.841	26	29	0.032	17	38
XP_624408	Oxidoreductase	G	0.151	35	20	1.000	28	27	0.032	38	17
XP_396922	Amine oxidase, isoform 1	G	0.151	35	20	0.421	23	32	0.056	18	37
XP_392970	Lon protease, isoform A	G	0.690	30	25	0.690	25	30	0.056	37	18
XP_623298	Tropomyosin 1, isoform A	G	1.000	27	28	0.841	29	26	0.056	18	37
NP_001035348	Troponin T	G	0.841	29	26	1.000	27	28	0.056	18	37
XP_623143	Pugilist, isoform A isoform 2	G	0.548	24	31	1.000	27	28	0.095	36	19
XP_623070	Pugilist, isoform A isoform 1	G	0.548	24	31	1.000	27	28	0.095	36	19
XP_624353	Aldo-keto reductase, isoform A isoform 1	T	0.421	23	32	0.008	15	40	0.310	22	33
XP_391994	Aconitase	T	0.841	29	26	0.016	39	16	0.690	30	25
XP_392997	Glutathione S-transferase 1-1 (GST class-theta)	T	0.421	23	32	0.032	17	38	0.690	25	30
XP_391843	Yippee interacting protein 2	T	0.222	21	34	0.056	37	18	0.548	31	24
XP_392722	Tat-binding protein-1	T	0.222	34	21	0.056	37	18	0.421	23	32
XP_001120471	3-hydroxyacyl-CoA dehydrogenase type II	T	0.310	22	33	0.095	36	19	0.690	25	30
XP_394469	Tubulin at 60D	T	0.008	40	15	0.310	33	22	0.421	32	23
XP_623673	Isocitrate dehydrogenase, isoform C isoform 2	T	0.016	39	16	0.151	20	35	0.222	34	21
XP_001122661	Glutamate oxaloacetate transaminase 1, isoform A	T	0.032	17	38	0.421	32	23	0.421	23	32
XP_394471	Tubulin, beta, 2	T	0.032	38	17	0.841	26	29	0.841	26	29
XP_624112	Vacuolar H+-ATPase 55 kD B subunit, isoform B	T	0.056	18	37	0.310	22	33	0.421	32	23
XP_393806	3-hydroxyacyl-CoA dehydrogenase, isoform A	T	0.056	18	37	0.421	23	32	0.690	30	25
XP_625027	Elongation factor 1-beta (EF-1-beta)	T	0.095	36	19	0.690	25	30	0.690	30	25
NP_001035323	Cytochrome P450 monooxygenase	G&T	0.056	37	18	0.008	15	40	0.008	40	15
XP_392479	14-3-3, isoform C isoform 1	G&T	0.421	32	23	0.032	17	38	0.008	40	15
XP_001122876	Hypothetical protein	G&T	0.841	26	29	0.095	36	19	0.008	15	40
XP_394645	HSC70-interacting protein, isoform A isoform 1	G&T	0.056	37	18	0.151	20	35	0.008	40	15
XP_624156	ATP synthase, isoform A	G&T	0.095	19	36	0.151	35	20	0.008	15	40
XP_395976	CG33257-PA	G&T	0.095	19	36	0.310	33	22	0.008	15	40
XP_001122872	Apidermin 2	G&T	0.032	17	38	0.016	39	16	0.016	16	39
XP_624041	Hexamerin 110 (Larval serum protein 2)	G&T	0.016	39	16	0.548	31	24	0.016	39	16
XP_392679	Dihydrolipoamide S-succinyltransferase	G&T	0.222	21	34	0.056	37	18	0.032	17	38
NP_001011578	Vitellogenin	G&T	0.095	36	19	0.095	36	19	0.032	38	17
NP_001011572	Transferrin	G&T	0.095	19	36	0.095	19	36	0.032	38	17
XP_397201	CG6459-PA	G&T	1.000	28	27	0.095	19	36	0.032	38	17
XP_392313	Tubulin at 56D, isoform B	G&T	0.032	38	17	0.310	33	22	0.032	38	17
XP_624781	60S acidic ribosomal protein P1 (RP21C)	G&T	0.548	24	31	0.008	15	40	0.095	36	19

Significant treatment and genotype effects (MWU test, n = 5, p<0.1, bootstrap verified cutoff). CL: control low pollen hoarding genotypes; KL: knockdown low pollen hoarding genotype; CH: control high pollen hoarding genotypes; KH: knockdown high pollen hoarding genotype; RS: rank sum; indicates which group showed a higher abundance. G: Genotype; T: treatment; G&T: Genotype and Treatment. See Table S1 for additional information.

However, abdominal VG and JH levels may not necessarily mirror hemolymph levels. Amdam *et al.* reported that VG expression in the abdomen and VG levels in the hemolymph are higher in bees of the high pollen genotype at 7–11 days of age [37]. In the same study, it was observed that while abdominal transcript and hemolymph protein levels correlate positively in the high pollen genotype, the relationship appears to be negative in the low pollen genotype. Taking into account the present data, we propose that VG is produced in the fat body and released into the hemolymph continuously in the bees of the high pollen genotype, while this is not necessarily the case for bees of the low pollen genotype. Our results indicate that the release of VG from the fat body into the hemolymph may play a crucial role in providing systemic effects of VG. In a study on Madeira cockroaches, *Leucophaea maderae*, it was previously found that the amount of VG already present in the hemolymph influenced the amount of VG released but not the one produced in the fat body [38]. It appears possible that bees of the low pollen genotype are more sensitive to this sort of feedback inhibition than bees of the high pollen genotype. How this would be achieved on a molecular level is currently unknown. We observed a trend (p = 0.0952) to lower VG levels in response to the *irs* knockdown [Figure 1D]. Overall, this finding is in line with previous data that reported a decreased trend of VG expression in response to an *irs* knockdown [23]. More comprehensive experiments, which include measurements of abdominal and hemolymph VG protein abundance, posttranslational modification state, as well as transcript level from the same bees, are needed to further investigate these connections. Another protein that was affected by genotype and knockdown was hexamerin 110 [Figure 1E]. Hexamerins are commonly described as energy storage proteins that can be used by the organism when the need arises, thus their abundance can be related to the nutritional status of the bee. It has been shown before that hexamerin expression in honeybee workers is dependent on the amount of protein consumed [39]. In a previous study on honeybee caste development, we showed a negative impact of downregulating *irs* expression on hexamerin 110 abundance. The tendency for a decrease of hexamerin 110 levels in response to an *irs* knockdown was found for both genotypes in the present study. However, the effect was only significant in the low pollen hoarding genotype. Another protein that showed a genotype-dependent knockdown response is a 14-3-3 protein with homology to yeast 14-3-3 epsilon [Table 1]. The levels of this protein were elevated in KH vs CH and in CL vs CH, but we detected no response to the knockdown in the low pollen genotype bees. The yeast homologue of this protein has been shown to associate with *irs* [40], and, although the function of this interaction remains unknown, it appears possible that increased levels of 14-3-3 epsilon increases the likelihood of its binding to *irs*. Thus, higher levels in KH vs CH could point to a mechanism in which higher levels of this protein compensate for lower *irs* levels and, in the case of higher levels in CL vs CH, to an inherent difference in insulin signaling-associated protein-protein interactions.

Based on observations in other species, we expected the *irs* knockdown to affect proteins involved in lipid metabolism. Indeed, we detected a genotype-specific knockdown effect on two 3-hydroxyacyl-CoA dehydrogenases (HADHs) [Table 1]. One of them (higher in KL vs CL) is the classical enzyme involved in fatty acid degradation, while the other is a type II HADH that may serve as a broad-spectrum oxidoreductase and can fulfill a variety of functions [41]. Higher levels of the classical HADH indicate a knockdown effect on lipid metabolism in the bees of the low pollen genotype and may point to an increased lipid turnover in this group.

Transferrin levels were significantly higher in the knockdown compared to controls in both genotypes and higher in CL vs CH [Table 1]. Transferrin is a protein that transports iron within the body and is thus directly involved in iron storage. In vertebrates, transferrin has been identified as a potent insulin antagonist [42], and its levels have been shown to be a predictor of hyperglycemia [43]. However, the molecular mechanisms behind these functions are unknown.

Two enzymes that may participate in the TCA (tricarboxylic acid) cycle showed knockdown-dependent differences in abundance: aconitase and dihydrolipoamide S-succinyltransferase (this enzyme may form part of the alpha-ketogluarate or the pyruvate dehydrogenase complex) [Table 1]. Both displayed lower abundance in *irs* knockdowns over the control for only the high pollen genotype; thus, it appears that the TCA cycle may be negatively affected by the knockdown in the high pollen strain. The isocitrate dehydrogenase identified here has the highest homology to an NADP+-dependent cytoplasmic isoform rather than to one that participates directly in the citric acid cycle and is thus not discussed in this context. The patterns in the high pollen hoarding genotype could reflect that reduced insulin-insulin like signaling (IIS) disturbs the TCA cycle akin to what has been described for diabetic rats [44], [45]. It should be noted that TCA cycle enzymes were found to be regulated in the opposite direction in nematodes (upregulated in individuals with reduced insulin signaling) [46]. This observation points to a possible life-cycle or species-specific impact of IIS on the TCA cycle.

We also identified two proteins that were influenced by the knockdown in both genotypes, albeit in different directions. The levels of a protein homologous to a cytochrome P450 monooxygenase (CYP), a family of enzymes involved in the metabolism of hormones and toxic compounds, were decreased in knockdowns of the low pollen hoarding genotype and increased in knockdown bees of the high pollen hoarding genotype compared to controls [Table 1]. In rat hepatocytes, the expression of a cytochrome P450 isoform was shown to be negatively correlated to insulin levels and also to the degree of insulin receptor phosphorylation [47]. Furthermore, in a comprehensive study on transcript levels from mice, flies, and nematodes, it was observed that many but not all proteins of the cytochrome P450 family were upregulated as a response to reduced insulin signaling [48]. Our finding of increased CYP levels in knockdown bees of the high pollen hoarding genotype [Figure 1F] with reduced insulin signaling capacities appears to be in agreement with these data. On the other hand, results from bees of the low pollen hoarding genotype show the opposite trend and thus indicate a genotype-specific response. The implications of insulin signaling for the expression of the various cytochrome 450 genes are complex and deserve further investigation. In summary, both treatment and genotype clearly affected the proteomic pattern in the abdomen of worker honeybees and provided us with insights into the molecular underpinnings of phenotypic differences in adult honeybees. This assessment was independently confirmed by a mixed-model ANOVA, which corroborated genotype, treatment, and/or interaction effects for 33 proteins, all of which had also been identified in the non-parametric analysis (data not shown). Next, we asked the question of how the abundance levels of abdominal proteins vary in each of the four phenotypically characteristic groups in a maturation-dependent manner.

### 2. Effects related to age, genotype, and knockdown of *irs* expression

In an independent experiment aimed at revealing age-related differences in the same four groups (CL, KL, CH, KH), we compared protein abundance at 9 and 11 days of adult life. We employed an approach that relies on isotope-labeling [49], carried out as previously described [50]. We used this approach rather than relying on spectral counting as in experiment 1, since labeling permits running different samples simultaneously, which should increase accuracy of quantification [51]. Since the following experiments consider a number of different variables, and this differential isotopic labeling method allows only for binary analyses, we had to select a sample that serves as a reference and can bridge and connect results from different groups. We chose 7-day-old bees as a reference in two different ways:

- We evaluated maturation-dependent changes within each of the four groups, using 7-day-old bees as reference. This procedure allowed for a non-parametric analysis in line with the procedures employed in the label-free proteomics experiment and detailed age-dependent changes in light of the metabolic starting point of each of the different groups (2.1.).
- After establishing age-dependent changes in all groups, we next decided to perform a complete analysis including genotype, treatment, and age as factors. To this end, we used 7-day-old bees from the low pollen genotype control group as a reference for all measurements and a mixed-model ANOVA for data analysis (2.2.).

#### 2.1 Age-dependent plasticity

The comparisons between 9- and 11-day-old bees were performed for all groups individually, using 7-day-old bees of the four different groups as separate reference points. Non-parametric Mann-Whitney U tests (n = 4 per group, p≤0.1, bootstrap verified cutoff) were used to evaluate statistical significance.

The largest number of differentially regulated proteins was observed in the control group of the low pollen genotype (CL). We thus discuss the results for this group first, and then relate the findings from the remaining groups to the data from this group.

**Figure 2:**
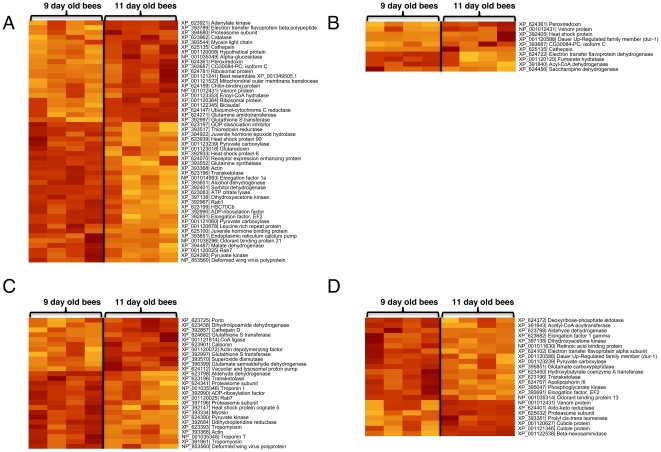
Age-related changes in protein abundance. Heatmaps of proteins that displayed abundance changes associated with age (Mann-Whitney U-test rank sum test, p<0.1, bootstrap corrected cutoff). Rows were clustered using Pearson correlation and complete linkage clustering. Rows represent proteins, columns represent samples. High values are distinguished by a red color, low values are yellow. A: Low pollen hoarding genotype control (CL); B: low pollen hoarding genotype knockdown (KL); C: high pollen hoarding genotype control (CH); D: high pollen hoarding genotype knockdown (KH).

Overall, we identified 50 differentially regulated proteins in the CL group [Figure 2A, Table S2, and Figure S4]. Levels of juvenile hormone are typically of higher abundance in foragers compared to nest bees [52]. Two potential regulators of JH levels, juvenile hormone epoxide hydroxylase (an enzyme involved in the degradation of JH) and a JH binding protein, were found at significantly lower levels in more mature bees. Since both juvenile hormone epoxide hydrolase and juvenile hormone binding protein are theoretically capable of reducing circulating JH titers [53], we propose that the abdominal fat body plays a major role in regulating JH levels during the behavioral transition from nest to forager bees through JH binding and degradation. However, it should be noted that a recent study found that JH epoxide hydrolase may only marginally contribute to JH degradation and is likely to play a more prominent role in lipid metabolism [54].

**Figure 3:**
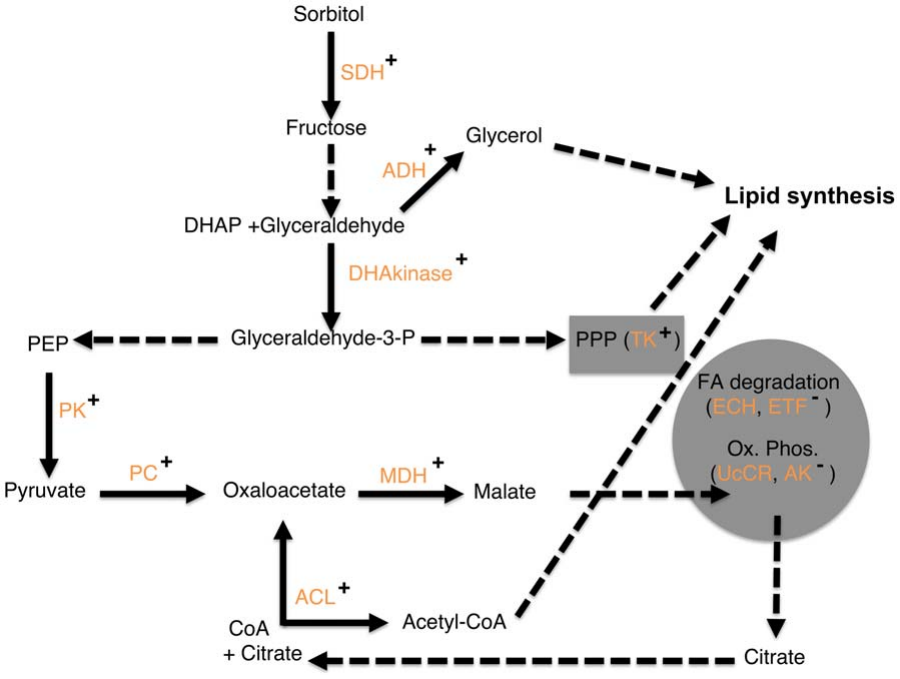
A model for possible metabolic connections in the honeybee abdomen. The model integrates findings on protein abundance from the low strain control bees and metabolic connections described in the literature (see text for details). Plus sign: higher abundance in 9-day-old vs 11-day-old bees. Minus sign: lower abundance in 9-day-old vs 11-day-old bees. Solid arrows: enzyme (shown in orange) showed age-dependent abundance differences. Dashed arrows: no evidence found in the present data but connections are supported by vertebrate studies. The model proposes a shift from lipogenesis to lipid degradation and higher oxidative phosphorylation with increasing age. SDH: sorbitol dehydrogenase; ADH: alcohol dehydrogenase; DHAkinase: dihydroxyacetone kinase; TK: transketolase; PK: pyruvate kinase; PC: pyruvate carboxylase; MDH: malate dehydrogenase; ACL: ATP-citrate lyase; ECH: enoyl CoA hydratase; ETF: electron trasfer flavoprotein; UcCR: Ubiquinol-cytochrome C reductase; AK: adenylate kinase; PPP: pentose phosphate pathway; FA degradation: fatty acid degradation; Ox. Phos: oxidative phosphorylation; PPP: pentose phosphate cycle; FA: fatty acid; grey circle: mitochondrium.

It is known that the fat body of honeybee foragers is substantially smaller than the one of nest bees, and that lipid levels drop even before the onset of foraging [16], [17]. We thus expected to see an image of this transition on the proteomic level. Overall, it appeared that carbohydrate and lipid metabolism change profoundly and in an integrated fashion during maturation [summarized in Figure 3]. Pyruvate carboxylase, cytosolic malate dehydrogenase, and ATP-citrate lyase have been implicated in lipogenesis in vertebrate adipose tissue [55], and it is tempting to suggest that they are metabolically connected in honeybees as well. Another enzyme, transketolase, is central to the interconversion of carbohydrates in the pentose phosphate cycle, thereby linking carbohydrates to lipid metabolism [56]. We found additional evidence for the involvement of carbohydrate and lipid metabolism in honeybee maturation among the proteins that were found at higher abundance in 11-day-old vs 9-day-old bees. In this category, we detected two proteins involved in fatty acid degradation (enoyl-CoA hydratase and ETF (electron transfer flavoprotein)), a key enzyme of oxidative phosphorylation (Ubiquinol-cytochrome C reductase), and an enzyme that interconverts ADP and ATP to maintain energy homeostasis and keep oxidative phosphorylation active (adenylate kinase). ETF couples fatty acid degradation to oxidative phosphorylation as it passes electrons to coenzyme Q, and subsequently these are transferred to ubiquinol-cytochrome C reductase [57]. Based on this data, it is tempting to speculate that younger bees utilize carbohydrates for the generation of lipids to a higher extent than older bees, in which the degradation of fatty acids becomes more important. These connections point toward a careful balance of carbohydrate and lipid metabolism during maturation. How could this be achieved? A prime candidate for regulating this network is the interplay between ChREBP (carbohydrate response element binding protein) and xylulose 5-phosphate as it is observed in vertebrates [58]. As levels of carbohydrate consumption increase, more xylulose 5-phosphate is produced in the pentose phosphate pathway, thus inducing nuclear translocation of ChREBP, where it aids in the transcription of genes involved in lipogenesis such as pyruvate kinase. This mechanism is intriguing, as it can link the nutritional carbohydrate status of the colony to the lipid levels in individual bees, which appear to be associated with the bee's social role. The varying abundance of the pentose phosphate pathway enzyme transketolase indicates the possibility for a positive feedback loop on transketolase expression, which would further stimulate the formation of xylulose 5-phosphate. A homologue of vertebrate ChREBP is present in the honeybee genome (data not shown), and future studies can investigate its postulated role in the onset of foraging.

In vertebrates, higher rates of oxidative phosphorylation and fatty acid oxidation can be accompanied by a higher production of reactive oxygen species [59], [60]. Fittingly, we detected higher levels of catalase, peroxiredoxin, and a glutathione S transferase (GST) in the more mature bees. Catalase and peroxiredoxin are known to be involved in the detoxification of hydrogen peroxide [61]. GSTs carry out diverse functions, usually related to the detoxification of harmful substances, such as peroxidized lipids and hydrogen peroxide [62].

Thus, we propose that honeybees shift from lipid synthesis to degradation during maturation. This shift is associated with an increase in the production of reactive oxygen species, which is matched by a higher abundance of enzymes that protect against these molecules.

As mentioned above, the fat body is known to lose lipids and volume before the bees start to forage [17], indicating a decrease of biosynthetic activity in this tissue. We observed lower levels of 5 proteins potentially involved in vesicle trafficking and membrane integrity (Rab 1 and 7, GDP dissociation inhibitor, ADP-ribosylation factor, and receptor expression enhancing protein) [63], [64] in 11-day-old bees. High capacities for vesicle transport could provide a framework for a higher biochemical activity. Thus, the data with lower capacities for vesicle transport in more mature bees may provide a molecular image of the visible decline of fat body metabolic activity during honeybee maturation [16].

As would be expected, if biosynthetic activity were indeed modulated during maturation, protein synthesis also appeared to be affected.

Elongation factors 1 alpha and 2 regulate the production of new proteins and are present at higher levels in less mature bees [65], while the two acidic ribosomal proteins P1 and P2, implicated in the specificity of protein synthesis [66], were found at lower levels in less mature bees. This finding indicates a higher but somewhat less precise protein synthesis in 9-day-old bees. On the other hand, two proteins involved in protein degradation, a proteasome subunit and cathepsin, were found at higher abundance in 11-day-old bees, indicating higher propensities for protein degradation in older bees. It would be expected that, when protein production is upregulated, proteins involved in protein folding should also be of higher abundance. This was indeed the case. Heat shock proteins (represented here by hsp 90, hsp 8 and hsc 70), which are often associated with protein folding [67], were more abundant in younger bees. The same held true for thioredoxin reductase and glutaredoxin, proteins that were recently implicated in protein folding in addition to their role in antioxidant defence mechanisms [68]. Finally, we found alpha glucosidase II, an enzyme that acts on 1,4 alpha glycosydic bonds and thus breaks multimeric sugars into monomeric glucose [69] at higher abundance in older bees. This enzyme is known to be of high abundance in the hypopharyngeal glands of foragers, where it could enable workers to convert nectar into honey [70], [71]. It also seems possible that it is used for the digestion of sucrose, as appears to be the case for its homologue in mosquitoes [72]. In any case, its changing abundance levels appear to be tightly linked to the life-history transition of the bee.

We next investigated abundance dynamics in the KL group for which we detected differences in 10 proteins [Figure 2B, Table S2, and Figure S4]. Among these, 4 (peroxiredoxin, cathepsin, CG30084-PC, and venom protein) were also identified in the low genotype control group, and the directionality of abundance was the same as in the CL group.

Two proteins involved in the degradation of lipids, acyl-CoA dehydrogenase and electron transfer flavoprotein dehydrogenase, were found to be of higher abundance in 9 vs 11-day-old bees. This contrasts findings for the control bees, as fatty acid metabolizing proteins were of higher abundance in the 11 vs 9-day-old bees in this group. Thus, as in experiment 1, it appears that the knockdown had an effect on the abundance dynamics of enzymes involved in fatty acid metabolism. Similarly, the only heat shock protein identified was found at higher levels in 11-day-old bees rather than in 9-day-old bees as in the control group.

Were the discussed trends also found in bees of the high pollen genotype?

We detected differences in 27 proteins in the CH group [Figure 2C, Table S2, and Figure S4], out of which 7 (pyruvate kinase, transketolase, rab7, the ADP-ribosylation factor, GST, actin, and a deformed wing virus protein) were also detected in control bees of the low pollen phenotype. The directionality of the abundance trends was the same between high and low pollen genotype bees in all cases. Other proteins that fit conceptually with the interpretations made for the low genotype control bees include another GST isoform, a superoxide dismutase (involved in the detoxification of the reactive oxygen species superoxide), and a heat shock protein (hsc 5). In contrast to the results in the low pollen genotype, two proteasome subunits were detected at higher levels in 9-day-old bees. This finding indicates the possibility of a difference in the dynamics of protein degradation between the two genotypes.

The last group consisted of *irs* knockdown bees of the high pollen hoarding genotype. In total, we detected 23 proteins with abundance variation between the 9 and the 11-day-old KH bees [Figure 2D, Table S2, and Figure S4]. Of these, 5 (transketolase, pyruvate carboxylase, dihydroxyacetone kinase, elongation factor 2, and venom protein) were also differentially expressed in the low strain control genotype bees. The trends for all of these proteins were the same in both groups. Other proteins again followed the mechanistic connections proposed on the basis of the low pollen genotype data. The abundance levels for elongation factor 1 gamma, deoxyribose-phosphate aldolase (an enzyme that, similar to transketolase, participates in the pentose phosphate cycle), and phosphoglycerate kinase (which functions in the same pathway as pyruvate kinase and pyruvate dehydrogenase), were higher in 9-day-old workers. Similar to the findings of the high pollen genotype control bees, a proteasome subunit was found to be of higher abundance in 11-day-old bees. Proteins involved in fatty acid degradation (ETF, hydroxybutyrate coenzyme A transferase, and acetyl CoA acyltransferase), were of higher abundance in 9-day-old workers - a trend that is comparable to the KL but not the CL group. In addition, proteins that could be involved in the transport of lipids, retinoic acid binding protein and apolipophorin III, were found at higher levels in younger bees. Thus, lipid metabolism is affected by downregulation of *irs* expression, and mobilization of lipid storage may occur more profoundly at a younger age in knockdown bees.

#### 2.2 Interaction effects related to age, genotype, and *irs* expression

In this experiment, we analyzed additional samples based on the labeling approach employed in 2.1 and relied on mathematical inference (see methods section for details) in order to complete a full-factorial design and enable insight into interaction effects between all factors. To facilitate the study of these complex effects, we employed a mixed-model ANOVA analysis rather than the previously used approach based on Mann Whitney U tests. In all, 37 proteins showed differential abundance between groups [Table 2 and Table S3].

**Table 2:** Age, genotype, and treatment effects as revealed by a mixed-model ANOVA.

Accession #	Similar to	A	A&T	A&G	T&G	A&G&T
XP_001120585	Cuticle protein	-	-	-	-	∧
XP_623197	GDP dissociation inhibitor	∧	-	-	-	-
XP_001120008	Hypothetical protein	-	-	-	-	∧
XP_623196	Transketolase	∧	∧	∧	-	∧
XP_001122872	Apidermin 2	-	∧	∧	∧	∧
XP_001121046	Succinyl-CoA synthetase	-	-	-	-	∧
XP_001120220	Niemann-Pick type C2	∧	∧	∧	-	∧
XP_392405	Heat shock protein	-	-	∧	-	∧
XP_392490	Retinoid and fatty acid binding protein	-	-	∧	-	∧
XP_624401	Aldo-keto reductase	-	-	∧	∧	∧
XP_625135	Cathepsin	∧	∧	∧	-	∧
XP_001120364	Ribosomal protein	-	-	-	-	∧
XP_001123353	Enoyl-CoA hydratase	-	-	∧	-	-
XP_001120025	Rab7	-	∧	-	-	∧
XP_001121060	Pyruvate carboxylase	-	-	-	-	∧
XP_391841	14-3-3 protein, isoform zeta	-	-	-	∧	-
XP_393294	Proteasome subunit	-	∧	∧	∧	∧
XP_393368	Actin	-	-	∧	-	-
NP_001014993	Elongation factor 1a	-	-	∧	∧	∧
XP_624456	Saccharopine dehydrogenase	-	∧	-	-	-
XP_623199	HSC70Cb	-	∧	∧	-	-
XP_623065	Succinate dehydrogenase	-	-	-	-	∧
XP_623962	Catalase	-	-	-	-	∧
XP_623798	Aldehyde dehydrogenase	-	∧	-	-	-
XP_392060	Phosphatidylethanolamine-binding protein	-	-	-	∧	-
XP_624102	Electron transfer flavoprotein	-	∧	-	-	∧
XP_623084	Aldehyde dehydrogenase	-	-	∧	∧	∧
XP_624662	Glutathione S transferase	-	-	-	-	∧
XP_624361	Peroxiredoxin	∧	∧	-	-	∧
XP_395851	Glutamate carboxypeptidase	-	∧	-	-	∧
XP_623095	Phospholipid-hydroperoxide glutathione peroxidase	-	-	∧	-	-
XP_624390	Pyruvate kinase	∧	∧	∧	-	∧
XP_392990	ADP-ribosylation factor	∧	∧	-	-	-
XP_623383	Hydroxypyruvate isomerase	-	-	∧	-	-
NP_001035314	Odorant binding protein 13	∧	-	-	-	∧
NP_001035346	Troponin I	-	∧	-	-	∧
NP_001035349	Alpha-glucosidase	-	-	∧	∧	-

∧: indicates a significant effect; -: indicates no effect; A: Age, G: genotype, T: treatment. This table shows significant age, genotype, and treatment effects as revealed by a mixed model ANOVA. See Table S3 for additional information.

Age was an influencing factor for 36 out of the 37 proteins: 33 were affected by genotype and 32 by treatment. All but one protein (GDP binding factor, influenced by age) were affected by more than one factor [Table 2].

Twenty-four proteins (about 65%) overlapped with the experiment that investigated age-dependent changes. The differences between the two experiments can be explained by two interconnected factors: 1. The present experiment contained more and different samples, which allows for the identification of previously unidentified proteins; 2. Two different statistical approaches were used in the analysis of the two experiments, which can result in lower numbers of identified proteins, even when additional samples are measured. Two proteins (about 5%) overlapped with proteins from experiment 1. This low overlap can be attributed to biological variation between experiments as well as to different extraction and quantification methods resulting in different subsets of proteins being extracted and sampled. It highlights the gain of information when using a diversity of different samples and approaches in tackling the proteome.

**Figure 4:**
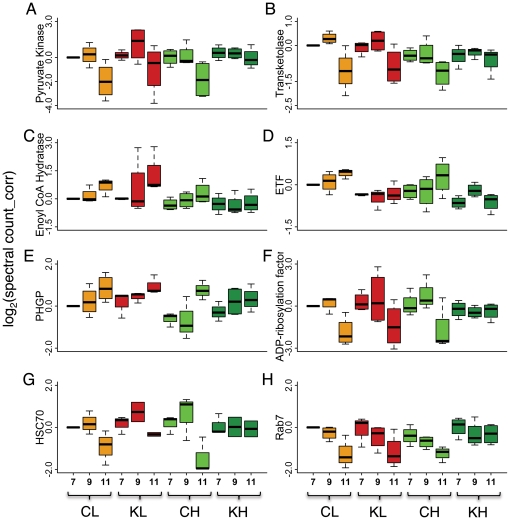
Proteomic indicators of the biosynthetic capacity of the abdomen are affected by treatment, genotype, and age. Boxplots (medians and 25–75 percentiles) of normalized protein abundance for A: PK (pyruvate kinase), B: TK (transketolase), C: ECH (enoyl CoA hydratase), D: ETF (electron transfer flavoprotein), E: PHGP (phospholipid-hydroperoxide glutathione peroxidase), F: ADP-ribosylation factor, G: HSC70, H: rab7. X-axes: Low pollen hoarding genotype control (CL), low pollen hoarding genotype knockdown (KL), high pollen hoarding genotype control (CH), high pollen hoarding genotype knockdown (KH). Numbers 7–11 refer to the ages (in days) of the bees. Normalized protein abundance: all values are relative to CL7 and log2-transformed. Y-axes: log_2_(spectral count_corr).

It is known that the two genotypes used in this study vary in maturation time [73], and that a decrease in insulin signaling (as can be caused by decreased levels of *irs* expression) can have a profound effect on developmental time, lifespan, and food choice in insects in general [58]–[60]. It is apparent from our results that age, treatment, and genotype interact to produce a complex proteomic pattern, which can ultimately affect phenotype during the days that typically precede the onset of foraging. We exemplify these complex effects on eight selected proteins that represent different protein categories. Overall, an age-dependent decrease in the abundance of proteins involved in lipid synthesis and carbohydrate metabolism (PK (pyruvate kinase), TK (transketolase); Figure 4A and B, respectively) as well as in vesicle trafficking and protein folding (ADP ribosylation factor, HSC70, Rab7; Figure 4F, G, and H, respectively) is accompanied by an increase in the abundance of proteins associated with lipid degradation (ETF, ECH (enoyl CoA hydratase); Figure 4C and D, respectively) and lipid peroxide removal (phospholipid-hydroperoxide glutathione peroxidase; Figure 4E). Expression trends for proteins involved in lipid metabolism follow our previous observations of a shift from lipid synthesis to increased lipid degradation with maturation. For the selected proteins depicted here, the high pollen genotype generally appeared to respond to downregulation of *irs* expression with a decreased age-dependent modulation of protein abundance [Figure 4]. The same pattern was observed for ETF but not for other proteins in the low pollen genotype. This observation suggests that some of the selected proteins are more prone to *irs*-dependent modulation in the high pollen genotype compared to the low pollen genotype. Nevertheless, the negative impact of the knockdown on ETF abundance in both genotypes corroborated an effect of the modulation of *irs* expression on proteins involved in lipid metabolism across genotypes. In order to assess whether the observed proteomic differences have an image on the metabolite level, we next measured lipid levels in the abdomen and glucose levels in the hemolymph.

### 3. Lipid and glucose levels vary with genotype and treatment

The aforementioned experiments showed that genotype, treatment, and age have an impact on the proteomic pattern during maturation. They suggest that the levels of proteins that are connected to lipid and carbohydrate metabolism are extensively remodeled in connection with the life-history transition of the worker honeybee. It is known that bees of the high and low pollen hoarding genotype show differences in their developmental speed and bees of the high pollen hoarding genotype start to forage earlier in life [24], [73]. We thus hypothesized that lipid storage, which is negatively correlated to in-nest tasks [15], [16], should be higher in bees of the low pollen hoarding genotype. In addition, *irs* expression and insulin signaling affect foraging behavior [23] and possibly the initiation of foraging [22] in honeybees. Importantly, *irs* is known to have an effect on lipid metabolism in fruit flies and mice. A knockout of the fruit fly homologue of *irs* leads to an increase in lipid levels [20]. Four different *irs* homologues are encoded in the mouse genome (IRS 1–4), the two most abundant of which are IRS-1 and 2 [74]. In analogy to data from fruit flies, it has been shown that knockouts of IRS-1 and IRS-2 lead to a change in the expression of enzymes involved in lipid metabolism and result in elevated lipid levels [21], [74], [75]. Given these findings in fruit flies and mice, we expected that a downregulation of *irs* expression in honeybees would similarly lead to an increase in lipid levels. Such a finding would further support a role for *irs* in the transition from nest to forager bee as this transition is closely associated with a change in lipid levels. In mice, IRS knockout can also influence glucose homeostasis. Knockouts of IRS-1 and 2 result in phenotypes typical of type 2 diabetes mellitus and insulin resistance with increased blood glucose levels at fasting and fed states (IRS-2) [18] and/or following an insulin tolerance test (IRS-1) [76]. Knockout of the lowly expressed IRS-4 leads to slightly decreased base blood glucose levels [19], while knockout of IRS-3 does not appear to have any effect on glucose homeostasis [77]. Based on these results and the fact that only one *irs* gene has been reported for the honeybee, we expected to find a modulation of hemolymph glucose levels in response to the *irs* knockdown.

**Figure 5:**
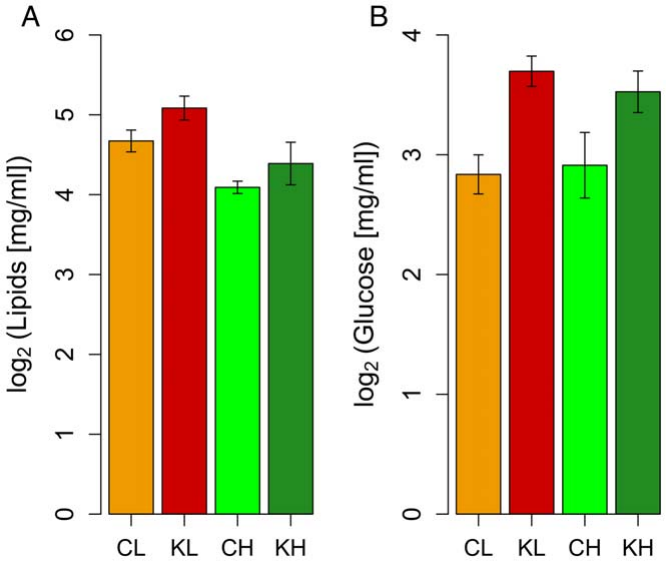
Abdominal lipid and hemolymph glucose levels at 11 days of age. A: overall, abdominal lipids were significantly affected by genotype (factorial ANOVA: genotype, F_(1,40)_ = 4.3019, 13.9294, p = 0.00059) and treatment (factorial ANOVA: treatment, F_(1,40)_ = 4.3019, p = 0.04455). B: overall, glucose levels in the hemolymph were significantly affected by treatment (factorial ANOVA, treatment, F_(1,59)_ = 14.89, p = 0.0002849) but not by genotype (factorial ANOVA, genotype, F_(1,59)_ = 0.006, p = 0.9395109). No significant interaction effects were observed for either lipid or glucose levels. Barplots represent means ± s.e. Orange: low pollen hoarding genotype control (CL); red: low pollen hoarding genotype knockdown (KL); light green: high pollen hoarding genotype control (CH); dark green: high pollen hoarding genotype knockdown (KH).

To test these ideas, we measured abdominal lipids and hemolymph glucose in 11-day-old bees of all four groups (CH: *gfp* control high pollen hoarding genotype; KH: *irs* knockdown high pollen hoarding genotype; CL: *gfp* control low pollen hoarding genotype; KL: *irs* knockdown low pollen hoarding genotype). We found that treatment had an effect on abdominal lipid (factorial ANOVA: treatment, F_(1,40)_ = 4.3019, p = 0.04455) and hemolymph glucose (two-factorial (factorial ANOVA, treatment, F_(1,59)_ = 14.89, p = 0.0002849) levels, and that genotype had an additional effect on lipids (factorial ANOVA: genotype, F_(1,40)_ = 4.3019, 13.9294, p = 0.00059). Interaction effects between *irs* knockdown and genotype were not significant. Barplots of the results [Figure 5] show higher levels of glucose and lipids in knockdowns and, as predicted, higher lipid levels in bees of the low pollen hoarding genotype.

In all, this data shows that the effect of an *irs* knockdown in honeybees on lipid levels mirrors the results for *irs* knockouts in fruit flies and mice, and that the two genotypes differ in their abdominal lipid levels in a way that can be predicted based on their differences in the onset of foraging. In addition, the higher glucose levels in response to the *irs* knockdown specifically resemble metabolic effects of an IRS-2 knockout in mice [18].

### 4. General conclusions

Our analysis showed that: 1.The abundance levels of enzymes involved in lipid metabolism are significantly affected by age, genotype and downregulation of *irs* expression; 2. Abdominal lipid levels are higher in the early-foraging genotype. They are also positively affected by a downregulation of *irs* expression in both genotypes. This result emphasizes the robustness of the metabolic response to the knockdown and is in line with the consequences of *irs* knockouts in fruit flies and mice. The increased lipid levels were accompanied by higher glucose levels, which are reminiscent of metabolic patterns in mice with disrupted IRS-2. Our data further suggest an age-dependent coordinated change in enzymes regulating lipid and carbohydrate metabolism, which is accompanied by a change in the abundance of proteins involved in antioxidative stress response, hormone metabolism, and protein turnover. In all, our results show how the proteome of a major tissue in honeybee workers is extensively remodeled preceding an important life-history transition. A transition from lipogenesis to lipid degradation in the fat body may help fuel the restructuring process in other body parts or occur concomitantly to those changes. In addition, it could aid in decreasing the weight of the bee before foraging flights. Furthermore, the fat body is capable of remotely influencing gene expression and hormone secretion in other tissues and to thereby influence overall organismal physiology and behavior. A change in the composition of the fat body as described in this study may thus play an important role in triggering behavioral and physiological changes in the worker honeybee associated with the transition from in-nest tasks to foraging. However, more information on fat body signaling during the transition will be needed in order to further clarify these connections.

## Materials and Methods

### Bee stocks

Selectively bred high and low pollen hoarding genotype *Apis mellifera* worker bees from two queens each were used in all experiments (see [30] for details on the selection program). Bees were kept and sampled in apiaries at Arizona State University (proteomics experiment 1) and the University of California, Davis. Queens were caged on a frame for 24 h in order to restrict the time of egg-laying. All frames were subsequently put into two colonies with wild-type bees as background. The night before emergence, frames were pulled from their respective hives and put in an incubator at 34°C with 45% relative humidity. Newly emerged high and low pollen hoarding genotype bees were injected intra-abdominally with either *irs* (insulin receptor substrate) dsRNA (double stranded RNA) or *gfp* (green fluorescent protein) dsRNA on the day of emergence. Thereafter, they were introduced into two replicate wild-type host colonies, and bees were retrieved when they were 7, 9, and 11 days old. The abdominal carcass (abdomen without digestive and reproductive systems) was used for RNA and protein extraction.

### Preparation of dsRNA and injections

The dsRNA synthesis and injection procedures were essentially performed as described previously [23].

The Qiaquick Gel Extraction Kit (Qiagen) was used to purify PCR products from 1% low melting agarose gels and the AmpliScribe T7 transcription kit (Epicentre Biotechnologies) was used for the production of dsRNA. Phenol∶choloform extraction was used to purify the resulting dsRNA and 3 µl of a 10 µg/µl solution (dsRNA in aqua dest.) were used for injections. The bees were chilled at 4°C for 5 min prior to injection, and dsRNA was injected into the hemolymph by inserting a needle dorsally under the penultimate tegument in the abdomen. Injections were conducted over two days and injected bees were marked with different colors. Injected bees of both strains were mixed and put into two wild-type *A. mellifera* colonies. Four sample types were generated: CH: high pollen hoarding genotype control, KH: high pollen hoarding genotype knockdown, CL: low pollen hoarding genotype control, KL: low pollen hoarding genotype knockdown.

### RNA extraction for a verification of the knockdown and protein extraction for proteomics experiment 1

Adult bees were collected, the digestive tract was pulled out of the abdomen, and the abdominal carcass was flash-frozen in liquid nitrogen. Samples were then stored at −80°C until further use. Individual carcasses were transferred into 500 µl of TRIzol® and extracted following the procedure provided by the manufacturer (Invitrogen). Thereafter, total RNA was treated with DNaseI (TURBO DNA-free™ Kit, Ambion) following standard instructions. For knockdown verifications, quantitative real-time PCR (qRT-PCR) was done in triplicate and analyzed with the comparative CT method [61] using the highest expression value as a reference. As before, *tubulin* at 56D CG9277-PB, isoform B (XM_392313) was chosen as a reference gene [78].

### Label-free proteomics

Equal amounts of samples dissolved in TRIzol® (see before) from three individuals were mixed and proteins were precipitated out of 200 µl of the trizol phase by using methanol∶chloroform precipitation [79]. Pellets were air-dried for 10–15 min before proceeding to protein digestion.

Proteins were redissolved in 50 µl of buffer containing 50 mM tris pH 8.5, 6 M urea, 2 M thiourea, 0.15 M NaCl, 1 mM CaCl_2_. Consecutively, 150 µl of the same buffer without urea and thiourea was added, samples were spun at 10,000 rcf for 2 min, and the supernatant was used for further analysis. The Bradford assay was used to determine protein concentration [80], and 40 µg per sample were subjected to digestion over night at 30°C with 1 µg of trypsin in digestion buffer (50 mM tris pH 8.5, 0.15 M NaCl, 1 mM CaCl_2_).

Peptide desalting was performed the next day as described before [70], [81].

LC-MS/MS analysis was essentially performed as described previously [70]. Dried peptides (from 10 µg protein) were dissolved in 5% acetonitrile, 2% TFA and used in a non-targeted LC-MS/MS analysis. Peptides were separated on a picofrit column (75 µm ID, New objective, Woburn, USA) using a 105 min gradient ranging from 95% A (0.1% formic acid, 99.9% H_2_O) to 80% B (0.1% formic acid, 99.9% acetonitrile) followed by a 15 min equilibration step. Peptides were eluted from the reversed phase µLC column directly into an LTQ mass spectrometer (Thermo, San Diego, USA), and the following settings were used: isolation window: 3 m/z, collision energy: 35, and activation time: 30 ms. MS^2^ spectra were recorded for the five most abundant peaks in each MS survey spectrum.

Forward sequences for trypsin, keratin, and the reverse sequences for all proteins were added to the *A. mellifera* sequence database retrieved from NCBI (http://www.ncbi.nlm.nih.gov/). The open source search tool OMSSA (version 2.0.0) [82] was used to match experimentally obtained spectra against the compiled database. The following filtering criteria were used: 0.8 Da fragment tolerance, 0.8 Da precursor tolerance, maximum of two missed cleavages, only tryptic sequences allowed, initially ten possible peptide hits per spectrum reported then filtered to one peptide hit per spectrum, variable modifications: methionine oxidation, deamidation of N and Q. Acceptance threshold for peptides: e≤0.1. The false discovery rate was determined to be 0% for proteins (at least two peptide hits were required for the identification of a protein).

The proteomics data from this experiment have been deposited at NCBI (http://www.ncbi.nlm.nih.gov/peptidome/repository/PSE144).

### Proteomics dependent on chemical labeling

As before, carcasses of three individuals were pooled for each sample. Samples were washed three times in ice-cold PBS before they were transferred to 2 mL safelock tubes (Eppendorf) containing a tungsten bead in 50 µl PBS and complete protease inhibitor tablet (Roche) at 8 times the suggested concentration. Samples were homogenized in a bead mill by shaking at 30 Hz for 5 min. Lysis buffer (100 µl of 1% NP-40, 150 mM NaCl, 20 mM Tris pH 7) was added to the samples and passed ten times through a syringe tipped with a 25 G needle, after which the samples were clarified for 10 min at 16,100 rcf at 4°C and the pelleted debris was discarded. Proteins were precipitated overnight by adding 1 mL ethanol (100%) with 20 µl of 3 M sodium acetate pH 5 and 20 µg of glycogen. Protein pellets were collected by centrifugation for 10 min at 16,100 rcf, briefly dried for 5 min in a vacuum centrifuge, and resolubilized in 30 µl in solubilization buffer (6 M urea, 2 M thiourea in 10 mM HEPES pH 8).

Protein concentration was determined in duplicates using the Coomassie Plus Protein Assay reagent (Pierce). Samples were aliquoted into 8 µg portions of 2 µl total volume and stored in −20°C until used.

Protein samples were reduced using 0.5 µg dithiothreitol for 30 min at 37°C, then carbamidomethylated by 2.5 µg iodoacetamide for 20 min. For overnight digestion, samples were mixed with 5% acetonitrile and 1 mM calcium chloride in 50 mM Tris pH 8 before adding 0.5 µg of modified trypsin. On the next day, 100 µl of sample buffer (1% trifluoroacetic acid, 3% (v/v) acetonitrile, 0.5% (v/v) acetic acid) was added to each sample and peptides were desalted as described above.

For isotopic labeling, peptides were resuspended in 10 µl of 500 mM sodium acetate pH 5 and mixed with one of three isotopologues of formaldehyde (10 µl at 200 mM) and one of two isotopologues of sodium cyanoborohydride (1 µl at 1 M): CH_2_O with NaCNBH_3_ (light-labeled), C^2^H_2_O with NaCNBH_3_ (medium-labeled), and ^13^C^2^H_2_O with NaCNB^2^H_3_ (heavy-labeled) [83]. The reaction was allowed to occur for two hours; samples were replenished with equivolumes of formaldehyde and cyanoborohydride after one hour. To quench the excess reagents, 10 µl of ammonium chloride (3 M) was added for 10 min, followed by 3-fold volumes of sample buffer for one hour. Samples to be compared were mixed in equal amounts (by protein amount) and purified as before by STAGE tips. Overall, four sample categories were measured: CH: *gfp* control, high pollen hoarding genotype; KH: *irs* knockdown, high pollen hoarding genotype; CL: *gfp* control, low pollen hoarding genotype; KL: *irs* knockdown, low pollen hoarding genotype. For the first labeling experiment, samples from all three timepoints (7, 9, and 11 days) of each sample group were labeled, mixed and measured (i.e. CL6/CL8/CL10, CH6/CH8/CH10, KL6/KL8/KL10, KH6/KH8/KH10 were measured separately. Four replicates were measured per trimethylated sample). The aim of the second experiment was to make all sample groups directly comparable to each other. CL6 was chosen as the reference sample and the following additional samples were measured: KH6/CL6, CL6/CH6, CH6/KL6 (all dimethylated) and KL6/KH6/CL6, KH6/CH6/CL6 (both trimethylated). The results from these measurements were used to compute remaining missing values.

All samples were eluted onto a 96-well autosampler plate, dried by vacuum centrifugation, and resuspended in sample buffer such that 4 µg was injected onto a reversed phase column (3 µm-diameter ReproSil-Pur C_18_, Dr. Maisch, Ammerbuch-Entringen, Germany), manually packed into a 15 cm-long, 75 µm-inner diameter fused silica emitter. Elution from the 1100 Series nanoflow high performance liquid chromatography system (Agilent) was directly coupled through a nanoelectrospray ion source (Proxeon, Odense, Denmark) to a linear trapping quadrupole-OrbitrapXL (LTQ-OrbitrapXL, ThermoFisher Scientific, Bremen, Germany) tandem mass spectrometer as described [84].

Raw data files were parsed through Extract_MSN.exe (ThermoFisher Scientific) and DTASuperCharge (v.1.37) using the default parameters to obtain peak lists. Using Mascot (v2.2), the peak lists were then read against a database compiled essentially as described above. The following parameters were used for the search: trypsin (allowing up to one missed cleavage), carbamidomethyl as a fixed modification, variable modifications of oxidation at methionine, and dimethylation at the N-termini and lysine ε-amino groups (+28.031300 – light, +32.05251 – medium, +36.0757 – heavy), 10 parts-per-million (ppm) peptide tolerance, 0.8 Da MS/MS tolerance, and ESI-Trap fragmentation characteristics. Results were saved in Peptide Summary format with the “Require Bold Red" option checked, applying a score cutoff corresponding to p<0.05, which is 27. MSQuant (http://msquant.sourceforge.net/) was used to semi-automatically extract chromatographic peak volumes (XIC) in the light, medium, and heavy isotopologues of each detected peptide. Peptides with an absolute calibrated mass error of >5 ppm were not further considered. To calculate the false discovery rate for proteins, a non-redundant list was compiled from all the samples and replicates. Moreover, the list was additionally confirmed using the same database used in the Mascot searches and the in-house Perl script 25inalist.pl. This script ensures that all protein hits have at least one non-redundant peptide match. As in proteomics experiment 1, proteins identified using only one peptide were eliminated and the false discovery rate for proteins was 0% (for proteins identified based on one peptide it was 0.6%). Peptide ratios were averaged in order to obtain a protein ratio. The data is accessible at the Honey Bee PeptideAtlas, March 2010 Build (https://db.systemsbiology.net/sbeams/cgi/PeptideAtlas/buildDetails?atlas_build_id=282).

### Glucose quantification

Hemolymph (1.0 µl) was collected into centrifuge tubes from individual honeybees 10 days after dsRNA injection. Samples were immediately transferred to liquid nitrogen and stored at −80°C until use. The Glucose Assay Kit (Sigma Aldrich) was used to measure the glucose concentration in hemolymph as described previously [23]. Absorbance was read at 340 nm on a spectrophotometer (Ultraspec 2100 pro, Amersham Biosciences). A standard curve using known amounts of glucose was used for quantification. Each sample was run in duplicate and average values were used for analysis (n = 17 (KH), 15 (CH), 15 (KL), 16 (CL)).

### Lipid measurements

Samples were collected from 11-day-old bees, transferred to a centrifuge tube, immediately flash frozen into liquid nitrogen, and stored at −80°C until use. Thereafter, samples were depleted of proteins using the deproteinizing kit from Biovision following manufacturer's instructions. Following protein removal, the samples were transferred to glass tubes and homogenized in 5 ml methanol∶ chloroform buffer (1∶2) overnight. Homogenized samples were filtered over glass wool, samples were dried to 2 ml (final volume) using a speed vac, and thereafter subjected to lipid analysis as described [17]. Absorbance was read at 525 nm on a spectrophotometer (Ultraspec 2100 pro, Amersham Biosciences). A standard curve using known amounts of pure cholesterol was used to calculate lipid amounts. Each sample was run in duplicate and average values were used for analysis (n = 11 (KH), 12 (CH), 10 (KL), 11 (CL)).

### Statistical analysis

R.2.10.1 was used for all calculations except where indicated otherwise. Expression data in high and low pollen genotype bees (*irs* RNAi and control) conformed to the assumptions of Levene's test and was analyzed with a factorial ANOVA.

Statistical analysis for proteomics experiment 1 was essentially conducted as described previously using non-parametric Kruskal Wallis and post-hoc Mann-Whitney U-tests [70] (n = 5 per group, p≤0.1, cutoff verified by a bootstrap procedure including 1000 iterations and sampling without replacement). At least two peptides per protein were required for considering a protein in the quantitative analysis. Further, quantification for proteins required the presence of a spectral count ≥3 in 3 or more of the 5 replicates of one group and the identification of at least 2 peptides in at least 4 out of 5 replicates in one group. Individual spectral counts were divided by the total spectral count to account for possible variations in total protein amount. Protein values were log_2_-transformed for HCA (hierarchical clustering analysis). Then, a pearson correlation matrix was calculated and complete linkage clustering was employed using the hclust function in the R stats package. The command prcomp in the stats package was used to calculate the principal components from the normalized spectral count matrix and the first two principal components, which explained >90% of the variance were plotted.

For the experiment monitoring maturation effects, non-parametric Mann-Whitney U rank sum tests were used to analyze the results analogous to the analysis in experiment 1 (n = 4 per group, p≤0.1, bootstrap verified cutoff). Only proteins that were present in at least three out of the four replicates of one group were considered. Sample values were z-transformed before analysis. Missing values were replaced first with +0.5 and next with −0.5 (±0.5 STDEV). P-values were calculated for both options and proteins were only retained if they were deemed to be statistically different between groups with both replacement options. For final calculations of p-values, +0.5 was used. To analyze interaction effects between all factors (age, treatment, genotype), we modified a standard approach used in the analysis of microarray datasets [85]. Raw values were log_2_-transformed and only proteins that were identified in all four replicates of at least one group were considered. Data were analyzed using a mixed-model ANOVA (mmANOVA) [85], [86], [87] implemented in SAS 9.1.3 (Cary, NC) with the following model: Y_abcd_ = treatment_a_+genotype_b_+age_c_+(treatment*genotype)_ab_+(treatment*age)_ac_+(genotype*age)_bc_+(treatment*genotype*age)_abc_+∼sample_d_+ε_abcd_ where a indexes the treatment (control or RNAi groups), b indexes the genotype (high or low pollen-hoarding line), c indexes the timepoint at which each sample was collected (7, 9, or 11 days), d indexes the sample within each group, and ε_abcd_ is normally-distributed error. The ∼denotes a random effect; all interactions were considered fixed effects. F-tests were calculated for each protein. P-values were corrected for multiple testing using a false discovery rate (FDR) adjustment in SAS using the PROC MULTEST procedure [88]. The significant proteins represent differences in relative protein abundance that are significantly associated with variation in treatment group, genotype, age, or their interactions.

Lipid and glucose data was log_2_-transformed and analyzed with a two-way factorial ANOVA calculating effects of treatment, genotype, and interaction. One abdominal lipid sample displayed a value more than three times the value of any other sample. A Dixon outlier test confirmed the outlier characteristic of this sample (p<2.2e-16) and it was thus excluded from any further analysis.

## Supporting Information

Figure S1 **RNAi-mediated downregulation of** ***irs*** **expression levels in workers of the high and low pollen hoarding genotype.** *Irs* transcript levels were significantly downregulated in adult high and low strain bees 6 days post *irs* dsRNA injection as determined by a factorial ANOVA (treatment: F(1,56) = 4.7652, p = 0.03325, n = 15). Bars represent mean ± s.e. *Irs* mRNA shown as the log-transformed relative quantities (RQ) of tubulin mRNA in individuals treated with either *irs* dsRNA or *gfp* dsRNA (control).
(TIF)

Figure S2 **The effect of downregulation of** ***irs*** **expression levels on the coefficient of variance.** The coefficients of variance (standard deviation divided by the mean, y-axis) were calculated for all quantifiable proteins. Bars represent mean ± s.e. A two-factorial ANOVA indicated a significant treatment but no genotype difference (treatment: F(1,580) = 11.7385, p = 0.0006555, n = 146). Light green: high pollen hoarding genotype control (CH); dark green: high pollen hoarding genotype knockdown (KH); orange: low pollen hoarding genotype control (CL), red: low pollen hoarding genotype knockdown (KL).
(TIF)

Figure S3 **Proteins with statistically significant changes in Experiment 1.** Comparisons were conducted by considering the effects of treatment – *gfp* control (C) or *irs* knockdown (K) in the genetic background of either high (H) or low (L) pollen hoarding 7-day-old bees. This Venn diagram illustrates the number of proteins with statistically significant regulation levels in the four relevant comparisons (CL/KL, KL/KH, KH/CH, CL/CH) and how many regulated proteins are shared among them.
(TIF)

Figure S4 **Proteins with statistically significant changes due to age in various genetic and treatment groups in Experiment 2.** This Venn diagram illustrates the number of proteins that demonstrate an age-associated change by comparing the abdominal proteomes of 7-day-old bees against 9- and 11-day-old bees. CH: *gfp* control, high pollen hoarding genotype; KH: *irs* knockdown, high pollen hoarding genotype; CL: *gfp* control, low pollen hoarding genotype; KL: *irs* knockdown, low pollen hoarding genotype.
(TIF)

Table S1 **Detecting protein abundance differences associated with genotype and treatment.** All proteins from label-free proteomics experiment 1 that matched the criteria for quantification are listed in this table. Tissue from 7-day-old bees was used and four groups were assayed: Low pollen hoarding genotype control (CL), low pollen hoarding genotype knockdown (KL), high pollen hoarding genotype control (CH), high pollen hoarding genotype knockdown (KH). See methods for further details.
(XLSX)

Table S2 **Detecting protein abundance differences associated with age.** All proteins from proteomics experiment 2 that matched the criteria for quantification are listed in this table. The experiment was aimed at detecting age-dependent changes in protein abundance in all four groups individually: Low pollen hoarding genotype control (CL), low pollen hoarding genotype knockdown (KL), high pollen hoarding genotype control (CH), high pollen hoarding genotype knockdown (KH). Samples of 7-day-old bees of each group served as a reference and statistical comparisons were thus possible between 9- and 11-day-old bees within each group. Each experimental group is organized in a different worksheet. The file also includes a table with all proteins that were found to show significant differences in abundance (worksheet: significant differences). See methods for further details.
(XLSX)

Table S3 **Detecting protein abundance differences associated with genotype, treatment, and age.** This table lists all significant age, treatment, genotype, and interaction effects detected in proteomics experiment 3 as determined by a mixed-model ANOVA (worksheet: significant differences). In addition, it contains the values for all significantly regulated proteins identified in this experiment. Compared to proteomics experiment 2, this experiment relied on additional measurements and mathematical inferring in order to allow for a direct comparison between all groups. 7-day-old bees of the CL group were used as a reference for all comparisons.
(XLSX)
