# Supplementary figures and images for: The Worker Honeybee Fat Body Proteome Is Extensively Remodeled Preceding a Major Life-History Transition
Source: PLoS One. 2011 Sep 28;6(9):e24794. doi: 10.1371/journal.pone.0024794 (PMC3182174; doi:10.1371/journal.pone.0024794)

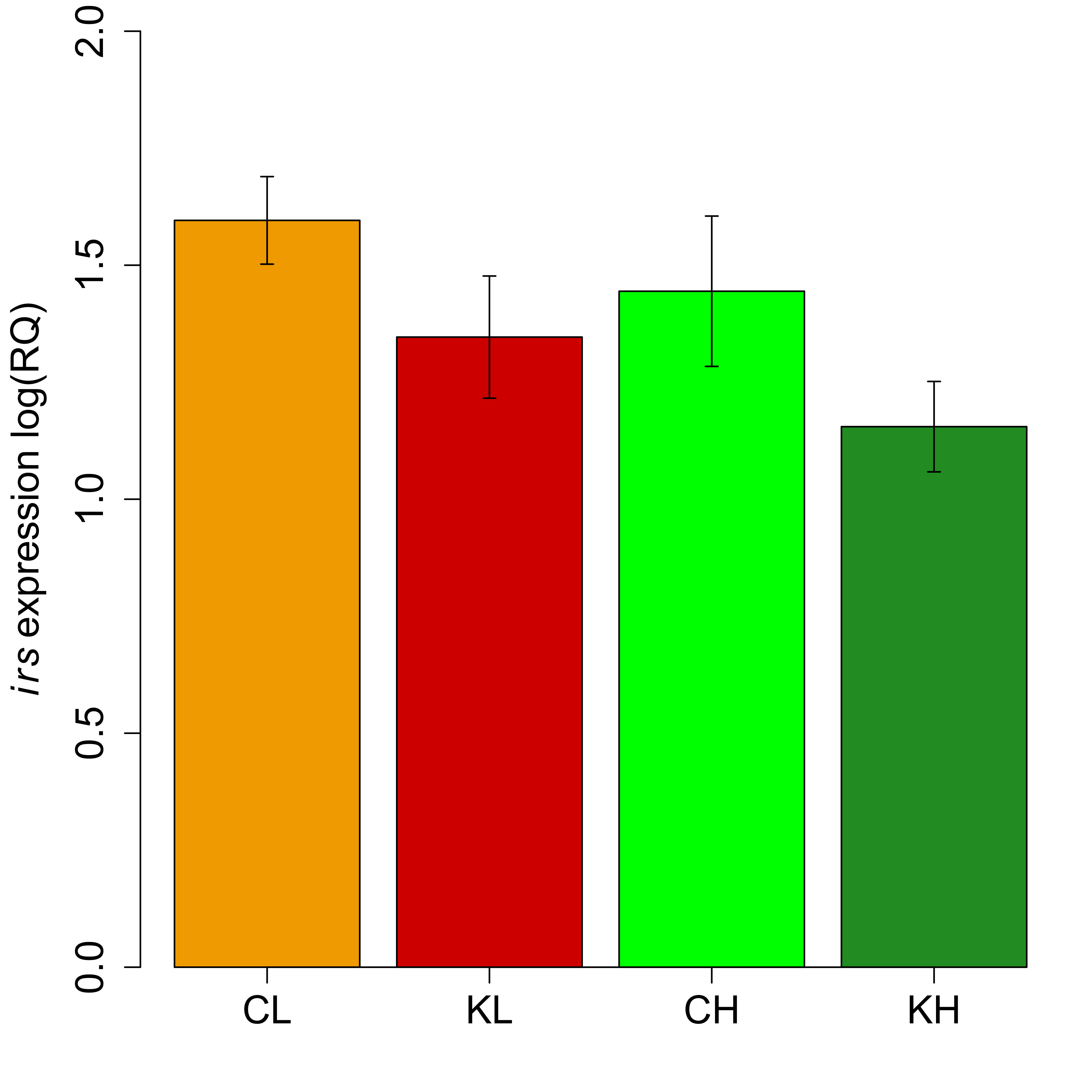

Supplement: Figure S1 — RNAi-mediated downregulation of irs expression levels in workers of the high and low pollen hoarding genotype. Irs transcript levels were significantly downregulated in adult high and low strain bees 6 days post irs dsRNA injection as determined by a factorial ANOVA (treatment: F(1,56) = 4.7652, p = 0.03325, n = 15). Bars represent mean ± s.e. Irs mRNA shown as the log-transformed relative quantities (RQ) of tubulin mRNA in individuals treated with either irs dsRNA or gfp dsRNA (control). (TIF) [file pone.0024794.s001.tif]

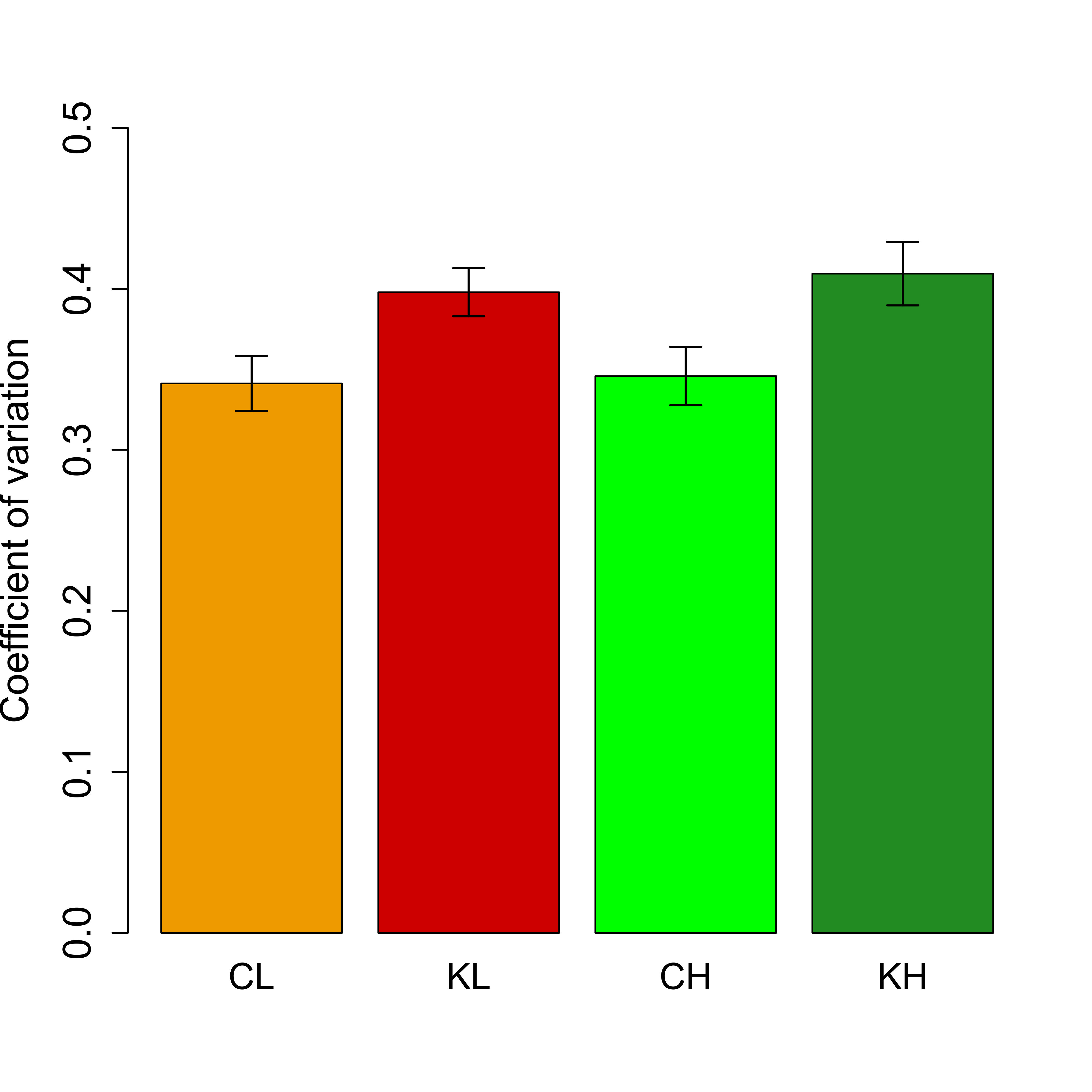

Supplement: Figure S2 — The effect of downregulation of irs expression levels on the coefficient of variance. The coefficients of variance (standard deviation divided by the mean, y-axis) were calculated for all quantifiable proteins. Bars represent mean ± s.e. A two-factorial ANOVA indicated a significant treatment but no genotype difference (treatment: F(1,580) = 11.7385, p = 0.0006555, n = 146). Light green: high pollen hoarding genotype control (CH); dark green: high pollen hoarding genotype knockdown (KH); orange: low pollen hoarding genotype control (CL), red: low pollen hoarding genotype knockdown (KL). (TIF) [file pone.0024794.s002.tif]

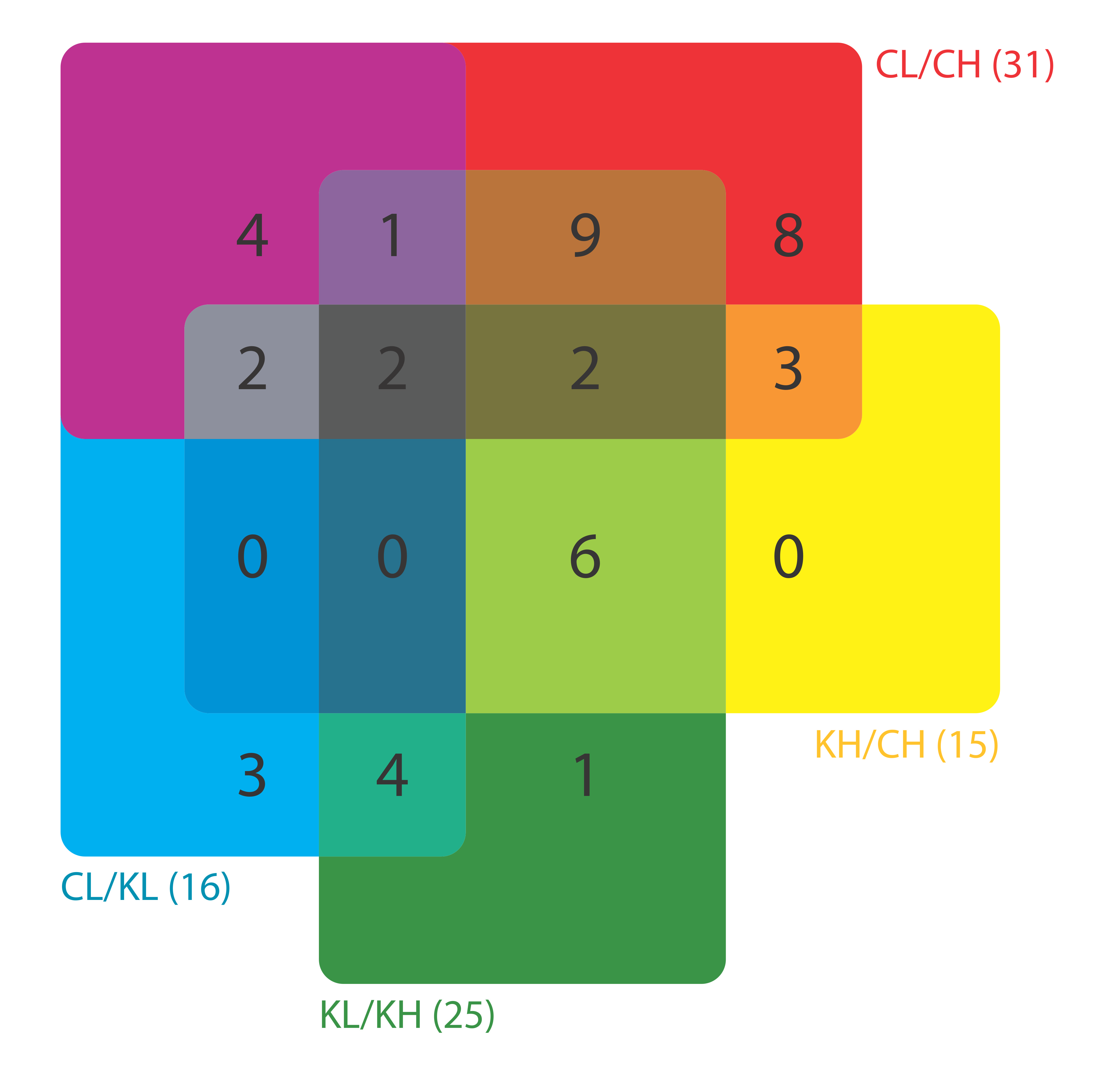

Supplement: Figure S3 — Proteins with statistically significant changes in Experiment 1. Comparisons were conducted by considering the effects of treatment – gfp control (C) or irs knockdown (K) in the genetic background of either high (H) or low (L) pollen hoarding 7-day-old bees. This Venn diagram illustrates the number of proteins with statistically significant regulation levels in the four relevant comparisons (CL/KL, KL/KH, KH/CH, CL/CH) and how many regulated proteins are shared among them. (TIF) [file pone.0024794.s003.tif]

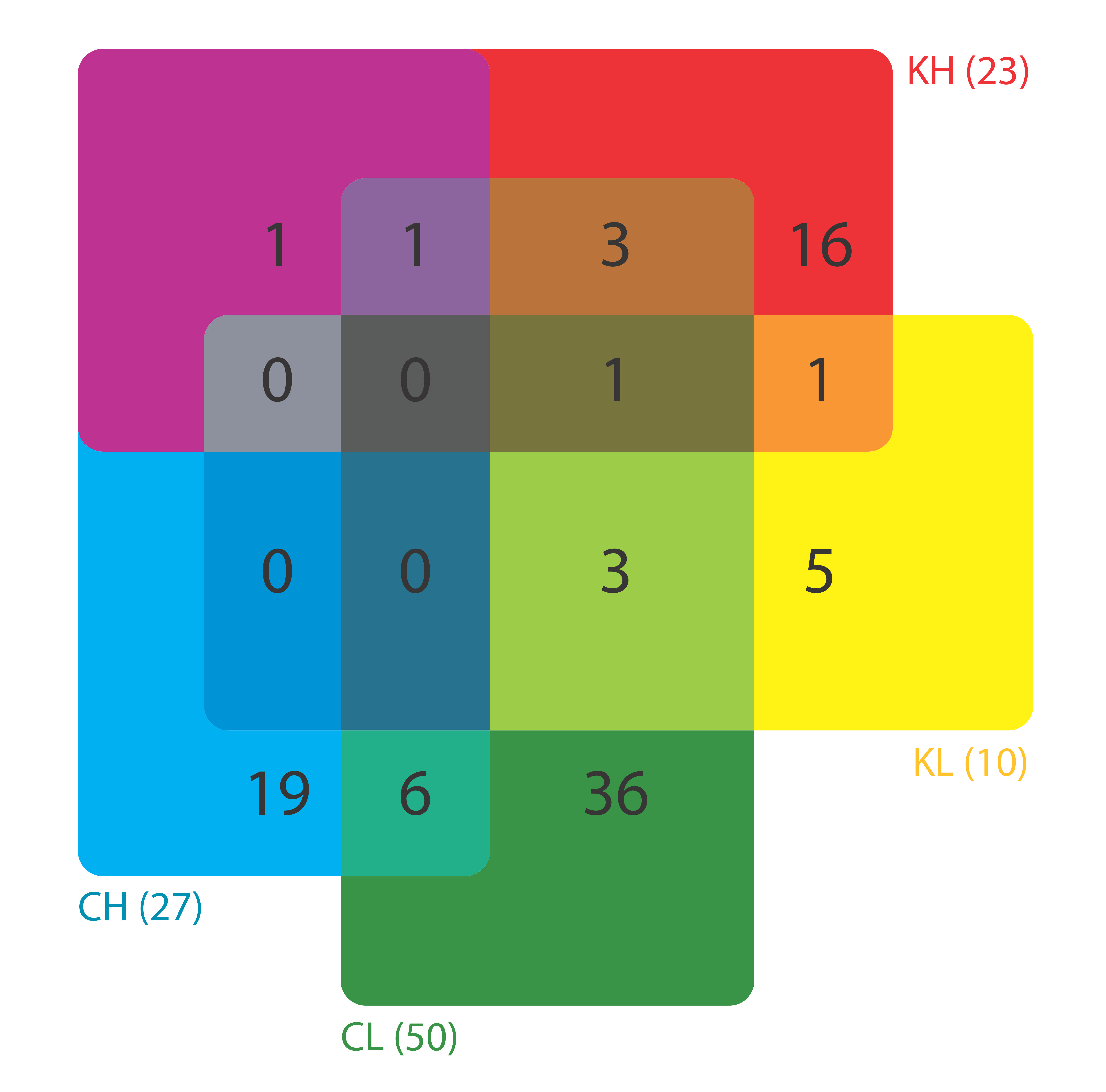

Supplement: Figure S4 — Proteins with statistically significant changes due to age in various genetic and treatment groups in Experiment 2. This Venn diagram illustrates the number of proteins that demonstrate an age-associated change by comparing the abdominal proteomes of 7-day-old bees against 9- and 11-day-old bees. CH: gfp control, high pollen hoarding genotype; KH: irs knockdown, high pollen hoarding genotype; CL: gfp control, low pollen hoarding genotype; KL: irs knockdown, low pollen hoarding genotype. (TIF) [file pone.0024794.s004.tif]
